# Maternal pre-pregnancy body mass index is associated with newborn offspring hypothalamic mean diffusivity: a prospective dual-cohort study

**DOI:** 10.1186/s12916-023-02743-8

**Published:** 2023-02-14

**Authors:** Jerod M. Rasmussen, Jetro J. Tuulari, Saara Nolvi, Paul M. Thompson, Harri Merisaari, Maria Lavonius, Linnea Karlsson, Sonja Entringer, Pathik D. Wadhwa, Hasse Karlsson, Claudia Buss

**Affiliations:** 1 Development, Health and Disease Research Program, University of California, Irvine, CA 92697 USA; 2 Department of Pediatrics, University of California, Irvine, CA 92697 USA; 3 FinnBrain Birth Cohort Study, Turku Brain and Mind Center, Department of Clinical Medicine, University of Turku, Lemminkäisenkatu 2, 20520 Turku, Finland; 4 Turku Collegium for Science Technology and Medicine (TCSMT), University of Turku, Turku, Finland; 5 Department of Psychiatry, University of Turku and Turku University Hospital, Turku, Finland; 6 Department of Psychiatry, University of Oxford (Sigrid Juselius Fellowship), Oxford, UK; 7 Turku Institute for Advanced Studies, Department of Psychology and Speech-Language Pathology, University of Turku, Turku, Finland; 8 Imaging Genetics Center, Mark and Mary Stevens Institute for Neuroimaging and Informatics, Keck School of Medicine, University of Southern California, Los Angeles, CA 90033 USA; 9 Centre for Population Health Research, Turku University Hospital and University of Turku, Turku, Finland; 10 Department of Clinical Medicine, Paediatrics and Adolescent Medicine, Turku University Hospital and University of Turku, Turku, Finland; 11 Department of Medical Psychology, Charité – Universitätsmedizin Berlin, corporate member of Freie Universität Berlin and Humboldt-Universität zu Berlin, Augustenburger Platz 1, 13353 Berlin, Germany; 12 Department of Psychiatry and Human Behavior, University of California, Irvine, CA 92697 USA; 13 Department of Obstetrics & Gynecology, University of California, Irvine, CA 92697 USA; 14 Department of Epidemiology, University of California, Irvine, CA 92697 USA

**Keywords:** Obesity, Pregnancy, Infant, Hypothalamus, Fetal programming

## Abstract

**Background:**

An extensive body of animal literature supports the premise that maternal obesity during pregnancy can alter the development of the fetal hypothalamus (HTH, a critical regulator of energy balance) with implications for offspring obesity risk (i.e., long-term energy *imbalance*). Yet, the relationship in humans between maternal overweight/obesity during pregnancy and fetal hypothalamic development remains largely unknown. Here, using an international (Finland and California, USA) multi-site diffusion tensor imaging (DTI) dataset, we test the hypothesis that maternal pre-pregnancy BMI is associated with newborn offspring HTH mean diffusivity (HTH MD, a replicable neural correlate of BMI in adults).

**Methods:**

HTH MD was independently quantified in two separate BMI-matched cohorts (up to class II obesity; BMI_Range_ = 17–35) using a high-resolution atlas-based definition of HTH. A total of *n* = 231 mother-child dyads were available for this analysis (*n*_Site,1_ = 152, age at MRI = 26.7 ± 8.1 days, gestational age at birth = 39.9 ± 1.2 weeks, *n*_M/*F*_ = 82/70, BMI = 24.2 ± 3.8; *n*_Site,2_ = 79, age at MRI = 25.6 ± 12.5 days, gestational age at birth = 39.3 ± 1.5 weeks, *n*_M/*F*_ = 45/34, BMI = 25.1 ± 4.0). The association between maternal pre-pregnancy BMI and newborn offspring HTH MD was examined separately in each cohort using linear regression adjusting for gestational age at birth, postnatal age at scan, sex, whole white matter mean diffusivity, and DTI quality control criteria. In post hoc analyses, additional potentially confounding factors including socioeconomic status, ethnicity, and obstetric risk were adjusted where appropriate.

**Results:**

The distribution of maternal pre-pregnancy BMI was comparable across sites but differed by ethnicity and socioeconomic status. A positive linear association between maternal pre-pregnancy BMI and newborn offspring HTH MD was observed at both sites ($\hat{\beta}$_Site,1_ = 0.17, *p*_Site,1_ = 0.01; $\hat{\beta}$_Site,2_ = 0.22, *p*_Site,2_ = 0.03) and remained significant after adjusting for cohort-relevant covariates.

**Conclusions:**

These findings translate the preclinically established association between maternal obesity during pregnancy and offspring hypothalamic microstructure to the human context. In addition to further replication/generalization, future efforts to identify biological mediators of the association between maternal obesity and fetal HTH development are warranted to develop targeted strategies for the primary prevention of childhood obesity.

**Supplementary Information:**

The online version contains supplementary material available at 10.1186/s12916-023-02743-8.

## Background

Childhood obesity is a major global public health challenge as it increases the likelihood of adverse obesity-related disorders in later life as well as their development at earlier ages [1] and with greater severity [2]. Further, primary prevention is crucial, because once established, obesity is challenging to overcome [3, 4].

Obesity is a highly multi-factorial phenotype [5], and the importance of the hypothalamus (HTH) in the regulation of energy balance is well established. Specifically, the HTH integrates afferent input from the periphery (e.g., the gastrointestinal tract), and it is sensitive to circulating satiety hormones (e.g., insulin, leptin and ghrelin) and nutrients (e.g., glucose, free fatty acids) to regulate feeding decisions [6–12]. A convergent body of imaging studies across species suggests that the typical structure (e.g., astrogliosis, axonal density, median eminence permeability) and function (e.g., leptin, insulin, and glucose sensing) of the human HTH is altered in the obese state. However, the temporal sequence of events leading to the observed differences in the HTH of individuals with obesity relative to those of normal-weight is largely unknown (i.e., whether altered HTH integrity is a cause or a consequence of the obese state).

Animal models suggest that the HTH exhibits developmental plasticity during the fetal period as it develops in the context of, and adapts to, variation in maternal overnutrition [13–20], which represents a state of excess maternal nutritional stores (e.g., adipocytes, circulating metabolic ligands) and their associated milieu (e.g., inflammation) from the perspective of the developing fetus. Fetal adaptations to maternal overnutrition have been shown to then influence postnatal metabolism and growth. Thus, the animal literature to date supports a framework wherein maternal obesity-induced variation in HTH structure and function may cause greater susceptibility to the postnatal obesogenic environment and may thus constitute a risk factor for obesity.

While evidence of the developmental plasticity of the *human* HTH in the context of interindividual variation in maternal overnutrition remains limited, it is supported by at least two recent studies. First, we recently reported on an association between maternal circulating saturated free fatty acid concentration (sFFA) during pregnancy and *newborn* offspring HTH microstructure indexed using diffusion tensor imaging (DTI)-based mean diffusivity (MD), that, in turn, was prospectively associated with early childhood adiposity [21]. Notably, higher maternal sFFA concentrations during pregnancy alter the development of the offspring hypothalamic microstructure in rodents [22] and are highly correlated with body mass index (BMI) in humans [23]. Second, maternal pre-pregnancy BMI was observed to be associated with offspring (aged 7–11 years) HTH function (blood flow, indexed using MRI-based arterial spin labeling) in response to oral glucose administration, that in turn, is associated with a longitudinal change in BMI [24]. However, prenatal and postnatal environmental effects in the latter study cannot be disentangled, as the outcomes were observed in middle/late childhood. Therefore, in the current study, we focus on the newborn brain, when the HTH has not yet been influenced by the postnatal environment. Furthermore, in the current study, we use maternal prepregnancy BMI as a surrogate measure of overnutrition that is associated with broad changes in maternal-placental-fetal metabolic, inflammatory, and endocrine biology [20] during pregnancy.

Based on these considerations, we set out to examine the prospective association between maternal pre-pregnancy BMI and newborn offspring HTH MD in two independent mother-infant cohorts. HTH MD is a replicable neuroimaging phenotype associated with BMI in human adults [25]. MD measures the mean diffusivity—i.e., the ability for unrestricted bulk water movement at the microstructural level, that is reflective of, but not specific to, several key microstructural properties including gliosis, axonal density, and median eminence integrity. Based on the direction of the previously replicated and reported BMI-HTH MD findings in adults—that either represent an HTH phenotype causative of energy imbalance (i.e., through altered function), or a consequence of energy imbalance (i.e., hypothalamic inflammation), or both—we hypothesized a positive association between maternal pre-pregnancy BMI and newborn offspring HTH MD. While our prospective study design cannot formally establish causality, it may help disentangle the temporal sequence of effects, as HTH MD was characterized shortly after birth, before much exposure to the postnatal obesogenic environment.

A prospective longitudinal study in two independent cohorts of mother-infant dyads followed from early pregnancy through shortly after birth was conducted to test the hypothesis that maternal pre-pregnancy BMI is positively associated with newborn offspring HTH MD. Maternal pre-pregnancy BMI was based on self-report (and confirmed by weight assessment in early pregnancy) and infant brain MRI scans were acquired shortly after birth to quantify HTH MD. Associations of maternal pre-pregnancy BMI with infant HTH MD were tested using multiple linear regression models that accounted for the effects of key covariates and potentially confounding factors.

## Methods

### Study population

**Fig. 1:**
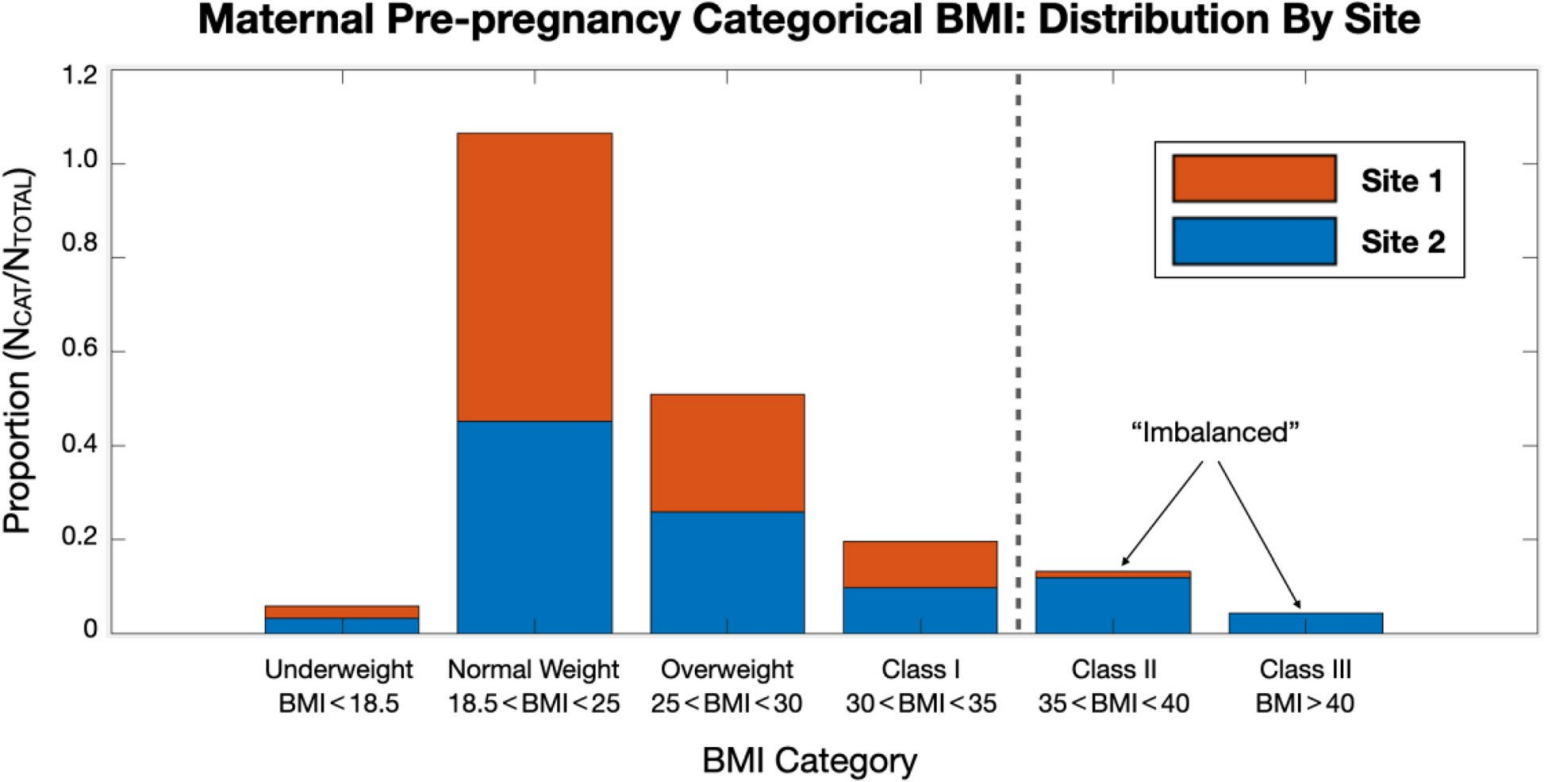
BMI distribution and matching by site. Sites were comparable in their BMI distribution after exclusion of class II obesity and above

Study populations at each respective site are described below, and they were similar with respect to maternal age, gestational age at birth, postnatal age at scan, and infant sex. Sites differed in their BMI distribution and therefore were stratified and matched by limiting participants to class I obesity and below (BMI < 35, Fig. 1) to improve compatibility between cohorts for partial replication, better estimate the effect size of pre-pregnancy BMI (i.e., matching variance), and minimize the effects of unobserved confounding variables (e.g., site differences in complications associated with class II obesity and above).

#### Site 1 specifics

Mother-child dyads were part of the FinnBrain Birth Cohort Study (www.finnbrain.fi, subsequently referred to as “site 1”), established in 2011 in South-West Finland in the area of Turku and Åland Islands to prospectively investigate effects of early life stress on child brain development and health [26]. Families were recruited to the study between 2011 and 2015. The present analysis considered 172 mother-child dyads (*n* = 152 after BMI-matching, see below) with an available offspring brain MRI scan shortly after birth (entire birth cohort *N* = 3808). The recruitment took place in the maternity clinics in Turku during the first trimester ultrasound visit at the gestational week 12, via a personal contact by the research nurse. Inclusion criteria for the enrollment were (a) a verified pregnancy and (b) sufficient knowledge of Finnish and/or Swedish (the official languages in Finland). Exclusion criteria for the neonates were as follows: occurrence of any perinatal complications with potential neurological consequences (e.g., hypoxia); less than 5 points in the 5 min Apgar score; previously diagnosed central nervous system anomaly or prior clinical MR scan at peripartum due to clinical indications. All infants in the imaging sample analyzed here were born near full-term (≥ 36 weeks of gestation) and weighed more than 2500 g at the time of birth. Families were provided oral and written information about the study, and the parents provided written consent to participate on behalf of their baby. The study was conducted in accordance with the Declaration of Helsinki, and it was approved by the Ethics Committee of the Hospital District of Southwest Finland (15.3.2011 §95, ETMK: 31/180/2011).

#### Site 2 specifics

Mother-child dyads were part of a prospective cohort study at the University of California, Irvine’s Development, Health and Disease Research Program (subsequently referred to as “site 2”). This study was designed to investigate the effects of maternal conditions during pregnancy on offspring development. The study enrolled pregnant women attending antenatal care at clinics affiliated with the UCI Medical Center in Orange County, California, between 2011 and 2015. The present analysis considered 94 mother-child dyads (*n* = 79 after BMI-matching, see below) enrolled between March 2011 and December 2013 with an available offspring brain MRI scan shortly after birth (entire birth cohort *N* = 253). Exclusion criteria were as follows: mother less than 18 years of age; non-singleton/intrauterine pregnancy; diabetes; maternal use of psychotropic medications or systemic corticosteroids during pregnancy; infant birth before 34-week gestation; and infant congenital, genetic, or neurologic disorder. The Institutional Review Board of the University of California, Irvine, approved all study procedures, and all parents provided written, informed consent (UCI IRB: #2009-7251).

### Pre-pregnancy body-mass-index (BMI) and covariate data collection

Our a priori covariate selection was based on theoretical considerations and evidence in the available literature of possible associations with either only the outcome of interest (to improve model precision) or with both the outcome and primary predictor of interest (to address potential confounding). Site 1 maternal pre-pregnancy BMI was based on a combination of self-reported and recorded measurement (source method is not specified at the individual level) abstracted from the widely used Medical Birth Register (Finnish Institute for Health and Welfare, Finland). At site 2, maternal pre-pregnancy BMI was based on maternal self-report and verified using height and weight measurements taken at the first pregnancy visit (*r* = 0.99). Previous reports have shown the high agreement between early pregnancy self-report and lab-based measures of BMI [27, 28] and thereby support the validity of self-report as a reliable indicator of maternal BMI. Gestational age at birth was determined by best obstetric estimate with a combination of last menstrual period and early uterine size and was confirmed by obstetric ultrasonographic biometry before 15 weeks using standard clinical criteria at both sites. Infant sex was abstracted from the medical record and infant scan age recorded on the day of scan. Self-report maternal education (level 1: high school/vocational school/test equivalent or less, level 2: associate’s degree, level 3: bachelor’s degree or higher) was harmonized across sites and used as a proxy for socioeconomic status (SES). Obstetric risk (OB risk) was harmonized across sites and consisted of a binary value indicating the presence of one or more of the following risk factors extracted from the medical record: hypertension, diabetes, severe anemia, severe infection, or vaginal bleeding. Maternal education was assessed by self-report at the first study visit (approximately the first trimester). Race and ethnicity were collected by self-report but only considered in the site 2 analyses (two-level categorical variable: Hispanic/non-Hispanic) due to ethnic homogeneity (100% White) of the site 1 (Finnish) sample. The rationale for the two-level categorical split was based on reporting as mandated by the US National Institutes of Health (NIH) and chosen to reflect the demographics of the cohort and provide the necessary cell sizes for a reliable statistical estimate of effects. Specifically, the sample sizes for the reported race categories (representative of the Southern California demographic) were considered insufficient (American Indian or Alaska Native *n* = 3; Black *n* = 3; Asian *n* = 8) to include in analyses at site 2, whereas stratification by reported ethnicity was relatively balanced (Hispanic *n* = 38; Non-Hispanic *n* = 41).

### MRI assessments

Data collection procedures broadly overlapped across sites in that they were performed on a single vendor (Siemens) and field strength (3 Tesla) with identical spatial resolution, but differed slightly in contrast and angular resolution, as described below.

#### Site 1 specifics

Newborn MRI scans were acquired during natural sleep using a 12-channel head-receive coil (Siemens 3T Magnetom Verio). Infants were fed with breastmilk or formula until they slept and then gently swaddled into a vacuum mattress. Personnel observed the scanning procedure through the window of the control room and interrupted scans in the case of the infant waking up. Images were acquired using a single-shell (*b* = 1000 s/mm^2^) 96-direction diffusion weighted protocol divided into three segments and previously confirmed to have good intra-scan test-retest reliability (EPI, TR/TE = 8500/90 ms, spatial resolution = 2 mm isotropic) [29].

#### Site 2 specifics

Newborn MRI scans were acquired during natural sleep using a 12-channel head-receive coil (Siemens 3T Tim Trio). After feeding and soothing, infants were placed in a CIVCO beaded pillow (www.civco.com). This pillow covers the infant’s body and head, becomes rigid under vacuum, and provides a comforting swaddle, motion prevention, and hearing protection when used in conjunction with standard foam earplugs. Participants were monitored for heart rate and oxygen saturation via a pulse-oximeter attached to the foot. Images were acquired using a single-shell (*b* = 1000 s/mm^2^) 42-direction diffusion weighted protocol (EPI, TR/TE = 8900/83 ms, spatial resolution = 2 mm isotropic) [30].

### MRI data pre-processing

**Fig. 2:**
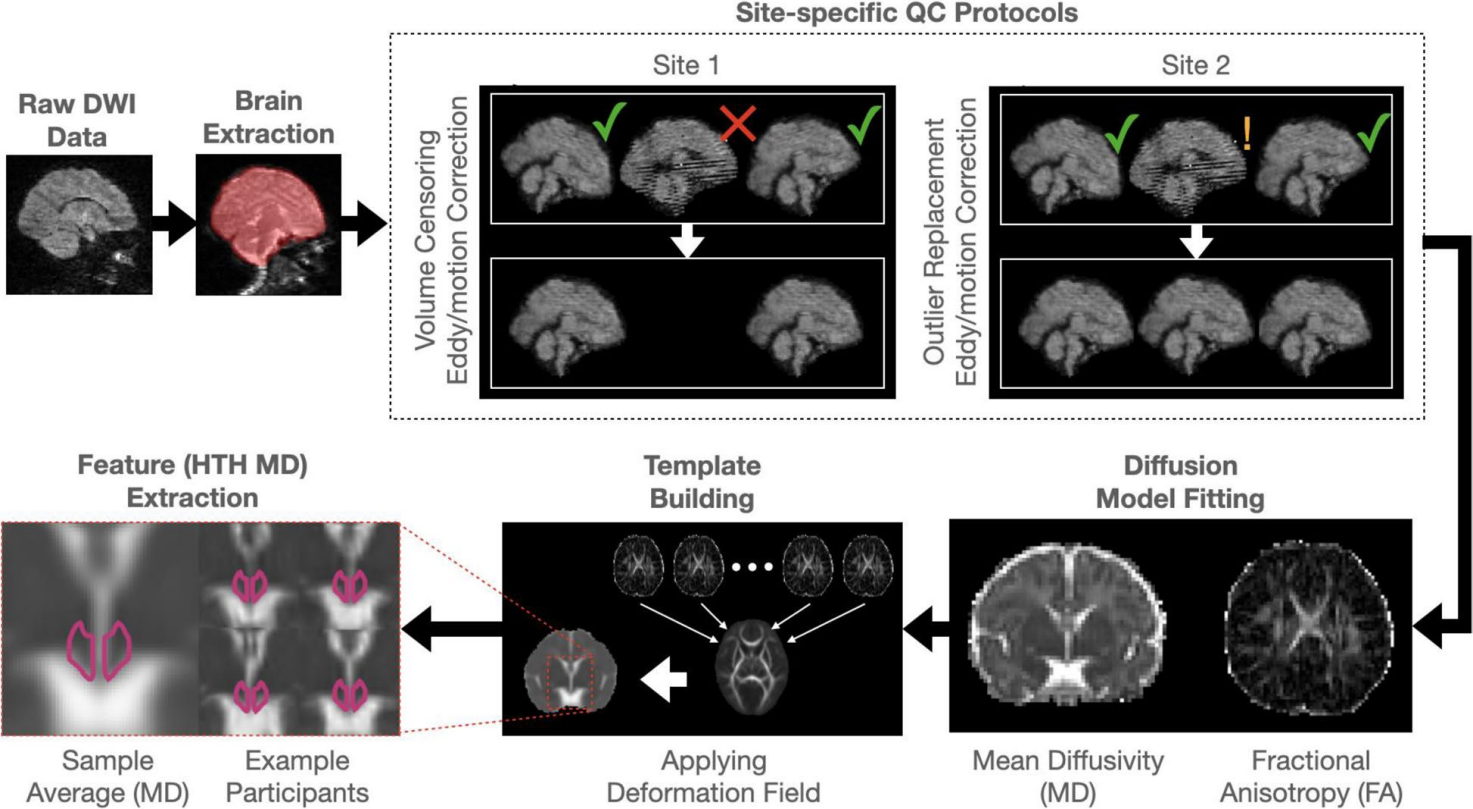
Site-specific image processing overview. Sites had broad overlap in image processing pipelines but differed in the approach to image quality control. Site 1 opted for censoring outlying volumes prior to eddy current and motion correction (upper fork), while site 2 opted for an integrated approach including the replacement of outlying volumes (lower fork)

Data pre-processing pipelines for time series correction were developed independently and performed by respective data collection sites prior to the initiation of the current study (see Fig. 2 for overview). In brief, while they were broadly overlapping (FSL’s *eddy* for motion and eddy current correction, *dtifit* for diffusion parameter fitting, *flirt* for spatial normalization, and image derived phenotype extraction based on a high-resolution HTH mask), they differed in approach to quality control (QC). Specifically, site 1 used a prospective data censoring approach and site 2 used a retrospective data replacement/correction approach with statistical adjustment for potential confounding, as described below. Brain masks were generated using the participant average *b* = 0 s/mm^2^ volume (site 1: range = 2–8 b0 volumes available, median = 5, site 2: 3–7 b0 volumes available, median = 7). Following time series correction, all processing steps were harmonized for the purpose of this study. Scalar maps (fractional anisotropy [FA] and MD) were fit to the corrected time series using FSL’s *dtifit*. Spatial registration to a common template was performed using FSL’s TBSS [31] procedure via *fnirt* in a two-stage process beginning with the generation of a sample-based template followed by non-linear registration to the sample-based template in MNI space. The adult template was used to initialize the creation of the sample template and MNI space was used as a common reference space between the templates and the probabilistic HTH mask. Finally, individualized deformation fields were then retrospectively applied to MD maps and bi-lateral median HTH MD was extracted based on the application of a binary HTH mask [32]. The binary mask was thresholded at a value of 0.6 and median statistics were used to minimize the potential impact of partial volume effects. Qualitative assessment of registration consistency in the vicinity of the HTH was used to verify proper alignment of data across both sites.

### Site 1

DWI data were visually QC’ed and datasets containing gross errors (e.g., excessive signal dropout or distortion) excluded from further analysis resulting in exclusion of *n* = 20 cases and a final dataset of *n* = 152. Brain masks were derived from averaged b = 0 volumes using FSL’s *bet*. Raw DWI data were QC’ed using DTIPrep and outlying volumes were censored from further analysis. For the purposes of the current study, we standardized the number of diffusion-encoding directions for each dataset by selecting directions in a manner that maximized angular resolution [29]. Censored datasets were then corrected for motion and eddy currents via FSL’s *eddy* [33].

### Site 2

DWI data were visually QC’ed and datasets containing gross errors (e.g., excessive signal dropout or distortion) excluded from further analysis. This resulted in exclusion of *n* = 2 cases and a final dataset of *n* = 79. Brain masks were derived from averaged *b* = 0 volumes using FSL’s *bet*. Data were corrected for motion, signal drop-out, and eddy current, including outlier slice detection and replacement using a Gaussian Process prediction via a non-parametric approach, and slice-to-volume correction. Data quality measures were extracted using FSL QUAD [34], screened for extreme outliers, and retained for use as a potentially confounding factor (mean framewise displacement [FD]).

### MRI data processing

#### Statistical analysis

Linear regression models were used to examine the relationship between maternal pre-pregnancy BMI and offspring infant HTH MD by first using a parsimonious regression analysis based on known or likely associations with infant HTH MD (Eq. 1), followed by a post hoc “full” model accounting for additional confounding factors where relevant (e.g., ethnicity). An additional interaction model testing for a sex-specific effect of maternal pre-pregnancy BMI on HTH MD was also considered by adding a pre-pregnancy BMI x offspring sex term (Additional file 1: Table S1). The rationale for the a priori inclusion of whole brain white matter (WM) MD (binary mask, FA > 0.2 on the constructed TBSS template) and FD is based on the premise that associations should be present above and beyond associations between maternal obesity and offspring global MD (e.g., non-linear age-related variation not accounted for by linear models) and limit noise-induced variation, respectively. Further, adjustment for WM MD allows drawing conclusions regarding the specificity of the association between maternal obesity and offspring HTH diffusivity over and above potential associations with global MD. However, a separate model excluding WM MD was tested and included as supplementary material (Additional file 1: Table S2) in order to aid the interpretation of effects without conditioning on WM MD. Because site 1 censors low quality data a priori and is of homogenous ethnicity, FD and ethnicity were not included in the site 1 parsimonious and full models, respectively. Equation 1 abbreviations are as follows in order of appearance: HTH_MD_ = newborn offspring hypothalamic mean diffusivity, pBMI = pre-pregnancy body mass index, GA = gestational age at birth, SA = postnatal age at MRI scan, sex = infant sex, WM_MD_ = white matter mean diffusivity, FD = framewise displacement.

1 $${HTH}_{MD,i}={\beta}_0+{\beta}_1\cdot {pBMI}_i+{\beta}_2\cdot {GA}_i+{\beta}_3\cdot {SA}_i+{\beta}_4\cdot {sex}_i+{\beta}_5\cdot {WM}_{MD,i}+{\beta}_6\cdot {FD}_i+{\varepsilon}_i$$

#### Data sharing plans

Guidelines for strengthening the reporting of observational studies in epidemiology (STROBE, https://www.strobe-statement.org/index.php?id=available-checklists) were followed and the checklist provided as part of the review process. Further, in accordance with the Material Design Analysis Reporting (MDAR) guidelines promoting access to underlying data and code, de-identified and unprocessed MRI data for site 2 used in the context of this study are publicly available through the National Institute of Mental Health Data Archive Collection #1890 (https://nda.nih.gov/edit_collection.html?id=1890). Unprocessed MRI data for site 1, de-identified pre-pregnancy BMI and associated (e.g., covariates) measures can be made available upon request and by completing a Data Use Agreement. Derived measures and statistical models used here are made publicly available via a Github repository (https://github.com/jerodras/bmi-hypothalamus.git).

## Results

### Study populations

A total of *n* = 231 pre-pregnancy BMI class-matched mother-child dyads were included for study. Pre-pregnancy BMI was available for all women whose offspring underwent MRI. The sub-sample of mother-child dyads (site 1 *n* = 152; site 2 *n* = 79) with maternal BMI and successful newborn imaging was representative (no significant differences in demographics, all *p* < 0.05) of the larger sample that did not include a successful imaging session (site 1 *n* = 172; site 2 *n* = 94).

### Descriptive findings

Maternal pre-pregnancy BMI was not significantly different (*p* < 0.05) between the matched samples at sites 1 and 2. Sites differed (*p* < 0.05) by maternal age at delivery (~2.4 year difference), gestational age at birth (~4 day difference), and ethnicity (site 1 homogenous non-Hispanic White), further supporting the stratified analyses approach used here, to avoid confounding by site. Sites were not significantly different based on maternal education, postnatal age at scan, sex, or OB risk (Table 1).

**Table 1:** Demographic information. The demographics of the sub-sample with successful imaging data were representative of the full sample that includes mother-child dyads without successful imaging (no sig. differences, *p* < 0.05)

	Site 1	Site 2
Maternal age [years (SD)]*	29.9 (4.4)	27.5 (5.5)
Maternal pre-pregnancy BMI continuous Maternal pre-pregnancy BMI class (%)	24.2 (3.78)	25.1 (4.0)
Underweight BMI < 18.5	*n* = 4 (2.6%)	*n* = 3 (3.8%)
Normal weight BMI 18.5–25	*n* = 95 (62.6%)	*n* = 42 (53.1%)
Overweight BMI 25–30	*n* = 38 (25%)	*n* = 25 (31.7%)
Obese BMI > 30 Maternal race and ethnicity (%)*	*n* = 15 (9.8%)	*n* = 9 (11.4%)
Hispanic	*n* = 0 0%	*n* = 38 (48.1%)
Non-Hispanic	*n* = 152,100%	*n* = 41 (51.9%)
Years of formal maternal education (%)		
Less than or equal to 12 years	*n* = 40 (27.2%)	*n* = 22 (27.8%)
Between 12 and 15 years	*n* = 49 (33.3%)	*n* = 30 (38.0%)
Greater than or equal to 15 years	*n* = 58 (39.5%)	*n* = 27 (34.2%)
Obstetric risk (yes/no)	*n* = 25 (16.5%)	*n* = 24 (30.3%)
Infant demographics		
Gestational age at birth [weeks (SD)]*	39.9 (1.2)	39.3 (1.5)
Postnatal age at scan [days (SD)]	26.7 (8.1)	25.6 (12.5)
Infant sex (*N* = male/female)	*n* = 82/70 (54/46%)	*n* = 45/34 (57/43%)

*A significant (*p* < 0.05) difference between sites

At site 1, mean infant HTH MD was 0.0017 ± 0.0001 mm^2^/s (S.D.); it was inversely associated with postmenstrual age at scan (partial *R*^2^ = 6.5%; *p* = 0.013) and did not differ by sex (*p* > 0.1). The presence and direction of these associations is consistent with those in the literature on early white matter developmental trajectories [35]. At site 2, mean infant HTH MD was 0.0013 ± 0.0001 mm^2^/s (S.D.); it was inversely associated with postmenstrual age at scan (partial R^2^ = 4.7%; *p* = 0.006) and did differ by sex (partial R^2^ = 4.0%; *p* = 0.01).

### The association between maternal pre-pregnancy BMI and offspring infant hypothalamic mean diffusivity across all sites

**Fig. 3:**
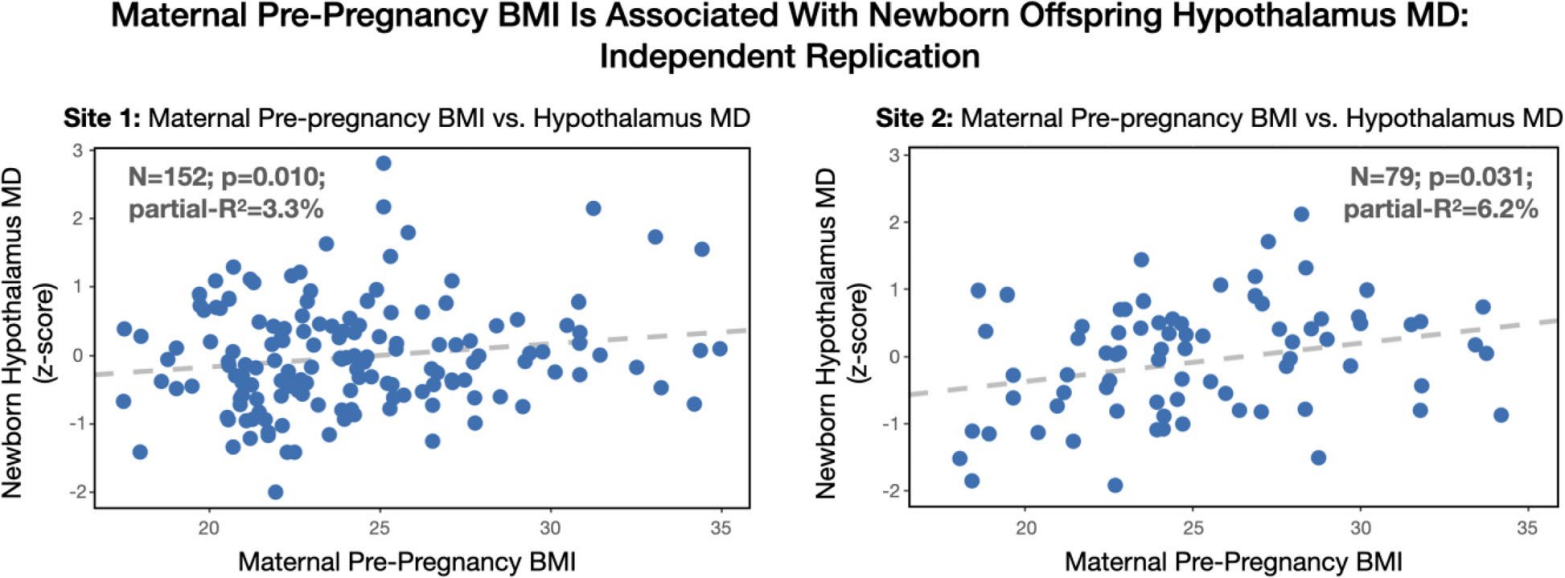
Maternal pre-pregnancy BMI and newborn offspring hypothalamic (HTH) mean diffusivity (MD) at two independent sites. The association between maternal pre-pregnancy BMI during pregnancy and newborn offspring HTH MD was significant at the two independent data collection sites

In both study populations, maternal pre-pregnancy BMI was positively associated with offspring infant HTH MD (Table 2; Fig. 3; Parsimonious regression model: *F*_5,152,Site 1_ = 19.00, *p* < 0.001, model adjusted-*R*^2^ = 37.1%; *F*_6,79,Site 2_ = 6.15, *p* < 0.001, model adjusted-*R*^2^ = 28.4%) over and above factors accounting for age-related growth (age and global variation in MD) and sex. Model residuals were normally distributed for both sites (Kolmogorov-Smirnov test *p* > 0.1). In addition, the association between maternal pre-pregnancy BMI and infant HTH MD also held in a more parsimonious model not adjusting for global variation in MD (Additional file 1: Table S1). In a separate model, there was no evidence for a sex-specific association between maternal pre-pregnancy BMI and offspring infant HTH MD at either site (*p* > 0.1; Additional file 1: Table S2). Maternal pre-pregnancy BMI remained significant when considering the potentially site-relevant confounding factors SES, ethnicity, and obstetric risk (Table 3; full regression model: *F*_7,152,Site 1_ = 12.37, *p* < 0.001, model adjusted-*R*^2^ = 35.9%; *F*_9,79,Site 2_ = 5.71, *p* < 0.001, model adjusted-*R*^2^ = 35.2%). Collectively, maternal pre-pregnancy BMI explained ~3–6% of the inter-individual variance in offspring infant HTH MD.

**Table 2:** Parsimonious model site comparison: maternal pre-pregnancy BMI and offspring hypothalamic (HTH) mean diffusivity (MD)

Maternal pBMI and offspring hypothalamic (HTH) mean diffusivity (MD): parsimonious model						
	Site 1 (***n*** = 152)			Site 2 (***n*** = 79)		
Independent variable	$\hat{\boldsymbol{\beta}}$	***R***^**2**^_***partial***_	***p***	$\hat{\boldsymbol{\beta}}$	***R***^**2**^_***partial***_	***p***
Maternal pre-preg. BMI	0.17	3.2%	*0.0107*	0.22	6.3%	*0.031*
Gestational age at birth	0.24	4.2%	*0.0109*	0.19	2.3%	0.192
Postnatal age at scan	0.46	14.7%	*< 0.001*	0.32	4.7%	0.063
Infant sex (male)	0.12	2.7%	0.0653	0.09	1.1%	0.363
White matter MD	0.59	28.5%	*< 0.001*	0.69	18.4%	*< 0.001*
QC (framewise displacement)	na	na	na	0.20	5.6%	*0.043*

*na* not applicable; the data were denoised by removing images with motion; see the “Methods” section for details

*Abbreviations*: *BMI* body mass index, *MD* mean diffusivity, *QC* quality control

**Table 3:** Full model site comparison: maternal pre-pregnancy BMI and offspring hypothalamic (HTH) mean diffusivity (MD)

Maternal pBMI and offspring hypothalamic (HTH) mean diffusivity (MD): full model						
	Site 1 (***n*** = 152)			Site 2 (***n*** = 79)		
Independent variable	$\hat{\boldsymbol{\beta}}$	***R***^**2**^_***partial***_	***p***	$\hat{\boldsymbol{\beta}}$	***R***^**2**^_***partial***_	***p***
Maternal pre-preg. BMI	0.15	3.5%	*0.0289*	0.26	9.4%	*0.009*
Gestational age at birth	0.21	3.2%	*0.0344*	0.17	2.2%	0.214
Postnatal age at scan	0.45	13.5%	< 0.001	0.21	2.6%	0.181
Infant sex (male)	0.13	2.5%	0.0608	0.13	0.7%	0.479
White matter MD	0.58	28.1%	*< 0.001*	0.64	18.8%	*< 0.001*
QC (framewise displacement)	na^a^	na^a^	na^a^	0.16	4.3%	0.085
Maternal education	0.02	1.7%	0.6771	0.27	8.7%	*0.012*
Hispanic (yes)	na^b^	na^b^	na^b^	0.31	10.5%	*0.005*
Obstetric risk (yes)	− 0.04	1.6%	0.5750	− 0.28	12.9%	*0.002*

*na*^a^, not applicable; the data were prospectively denoised by removing datasets with motion; see the “Methods” section for details. *na*^b^, not applicable; the ethnicity for FinnBrain participants was uniform—please see the “Methods” section and Table 1 for details

*Abbreviations*: *BMI* body mass index, *MD* mean diffusivity, *QC* quality control

## Discussion

The findings reported here demonstrate an approximately linear association between maternal pre-pregnancy BMI and offspring hypothalamic microstructure in two independent cohorts. Further, the observed association was consistent in direction and magnitude (overlapping 95% confidence intervals) at both sites and independent of sample-relevant potentially confounding factors. In addition, the generalizability of this association is supported by its apparent independence of site-specific demographics and choices in data preprocessing pipelines. Finally, because this finding is consistent with that in animal models demonstrating the plasticity of prenatal hypothalamic development in the context of maternal overnutrition in pregnancy, these observations provide evidence of a relationship that may be conserved across species.

A one standard deviation (SD) between-subject difference in maternal pre-pregnancy BMI was associated with a + 0.19 SD (95% CI: [0.11 0.27]) difference in infant HTH MD (HTH MD weighted average across cohorts). While the HTH MD phenotype used here is non-specific to the underlying microstructure, the observed direction of association (i.e., positive association between maternal BMI and the diffusion of water in the hypothalamus) is consistent with several microstructural phenotypes of obesity that reduce barriers to diffusion including reduced axonal outgrowth [22], gliosis [36], and/or structural changes to the median eminence [37]. In addition, the magnitude and direction of the maternal-fetal effect are consistent with reported estimates in the correspondence between within-subject BMI and HTH HD as observed in adults based on overlapping 95% confidence intervals, measured using similar techniques, and validated in two large independent samples [25]. This within-subject effect in adults has been argued to be a consequence of obesity-related neuroinflammatory processes with downstream implications on nutrient sensing and further weight gain thereby forming a positive-feedback loop. Thus, based on the observations made here that maternal BMI is positively associated with a newborn offspring neurophenotype that may represent suboptimal development (e.g., reduced axonal outgrowth, increased gliosis), we hypothesize that obesity-related maternal signals (e.g., nutritional, metabolic, endocrine, inflammatory factors) during gestation may contribute to altered nutrient sensing and bodyweight dysregulation in the offspring via the developing hypothalamus. However, an alternative interpretation that variation in hypothalamic microstructure is an inherited phenotype cannot be dismissed based on the current observational evidence. Future studies incorporating genetic risk profiles or sibling study designs with siblings being discordant for maternal prepregnancy obesity would be informative.

The U.S. Endocrine Society has argued that obesity should be conceptualized as a disorder of the energy homeostasis (balance) system [4], rather than simply arising from the accumulation of excess weight (i.e., above and beyond “move more, eat less”). Thus, the HTH nuclei, as the principal regulators of energy homeostasis within the central nervous system (CNS), are of obvious importance in the etiology of human obesity. This is further supported by literature suggesting that individual differences in the structural properties of the HTH are closely linked to variation in energy balance/imbalance across species [10, 25, 38]. In this context, the current work supports the HTH as a putative key target involved in the intergenerational transmission of maternal overweight/obesity during pregnancy via the programming of the CNS energy homeostasis system at birth. Moreover, because the effects of maternal states and conditions during pregnancy (e.g., maternal obesity) on the developing fetal HTH are ultimately mediated via gestational biology, future studies should focus on the underlying obesity-related variation in the intrauterine environment the fetus develops in and changes offspring HTH structure and function. A better understanding of the biological mechanisms involved will be necessary for informing future targeted interventions capable of breaking the intergenerational cycle of obesity.

The two cohorts analyzed here differed in maternal age, gestational age at delivery, and pre-pregnancy BMI. Cohort differences in maternal age and gestational age at delivery were in the expected direction [39–41], and the consistent association between maternal BMI and newborn offspring HTH MD at both sites suggests that this effect is robust to these site differences. With respect to maternal pre-pregnancy BMI as a predictor, cohort matching and stratification was used to better estimate the effect size of pre-pregnancy BMI and minimize the effects of other unobserved variables. However, the BMI-matched sample may have introduced bias, in that the exclusion of the higher BMI tail at site 2, but not site 1, could bias the selection towards low-susceptibility individuals at site 2 (under the assumption that site 2 may constitute a more obesogenic environment, as evidence by higher rates of insufficient physical activity and overweight/obesity risk in USA relative to Finland) [42]. While these differences may limit the direct comparison of effect sizes across sites, it does speak to the robust nature of the observation made here. That is, despite differences in obesity rates, other demographics, and data pre-processing strategies between sites, the association between maternal pre-pregnancy BMI and offspring HTH MD was consistent, suggesting this finding would be expected to generalize across other different populations.

Several potentially confounding factors were associated with offspring HTH MD above and beyond maternal pre-pregnancy BMI, and these warrant further discussion. Within site 2, OB risk, maternal education, and maternal ethnicity were all associated with HTH MD. Notably, OB risk and maternal education were not associated with HTH MD within site 1, suggesting that these are not replicable and/or robust observations in the context of this study (e.g., unobserved differences in assessment and/or differences in within site inter-individual variability). However, because site 2 demonstrated a substantially higher prevalence of OB risk, and because maternal education measures may reflect different socioeconomic pressures in different settings, these issues likely warrant further investigation. Relatedly, while significant at both sites, the effect sizes of postnatal age at scan and global measures of MD were markedly different across sites despite similar central tendencies. One explanation for this discrepancy could be differences in sample homogeneity (i.e., site 1 > site 2) where a larger proportion of variance is available for attribution to variance in maternal BMI. Alternatively, this observation could simply be due to differing approaches to handling low quality data wherein one excelled at removing measurement error relative to the other. Finally, the association between maternal ethnicity and offspring HTH MD suggesting lower neonatal HTH integrity among Hispanic neonates could not be tested at site 1 due to a high degree of ethnic homogeneity. However, because the direction of effect is consistent with higher rates of pediatric obesity among the Hispanic community [43], and because pathways underlying intergenerational transfer of obesity risk within the Hispanic community are understudied, this finding may warrant further attention.

The importance of identifying the role of BMI in the intergenerational transfer of obesity lies in its potential use as a screening tool for risk management (due to its ubiquitous use in clinical settings). In addition, from the perspective of a developing fetus, BMI is representative of the diverse metabolic gestational milieu (e.g., saturated fatty acids, glucose, insulin, leptin) in which the fetal brain develops. However, what BMI contributes in terms of representation, it lacks in specificity. That is, apart from generically informing policy and public awareness about the importance of maintaining a healthy pre-conceptional weight, this work provides limited insight into targeted interventions on the biological pathways of obesity risk transfer from mother to her offspring. For this reason, and because BMI alone is not always perfectly reflective of metabolic status (e.g., in metabolically healthy obesity), future efforts should aim to identify the specific ligands and conditions sufficient and necessary for the intergenerational transmission of obesity.

Weight stigma is among the most pervasive forms of discrimination within society, and is associated with adverse psychological (depression) and physiological (stress) conditions linked to increased weight [44] gain. It has been argued that one of the most effective approaches to hindering the perpetuation of weight stigma is the reinforcement of the concept within individuals and stigmatizing institutions that causes of obesity are not merely only a matter of will-power. Here, we focus on the hypothalamus, a central regulator of energy balance, as an entity that is not considered to be directly involved in “free-will,” per se. Clinically, hypothalamic obesity caused by damage to the hypothalamus (e.g., tumor excision) is reflective of a state of constant pressure towards excess energy intake with long term pressures often resulting in excessive weight gain. From this perspective then, because the premise supported here is one of intergenerational transmission of obesity risk via the hypothalamus, and because there is overlap between clinical hypothalamic obesity and “simple” obesity driven by the structural differences in the hypothalamus [45], this work further reinforces the anti-stigmatizing concept that many aspects of obesity are not simply a matter of “eat less, exercise more.”

Limitations of this study include the focus on a relatively healthy population, lack of gestational biological data, limited spatial resolution, and lack of specificity provided by the outcome (MD). Class I obesity represented roughly 10% of the study population and thus this study is likely underpowered to draw obesity-specific conclusions. To address this shortcoming, future studies would benefit from oversampling class I obesity and above to further identify consequences for fetal development specific to the obese condition. The current study did not collect or consider several developmentally important maternal ligands including the feeding-related hormones leptin [46] and ghrelin [47]. Further, future efforts should explore the influence of these and other placental, metabolic, inflammatory, and stress factors [20] and focus on their combined and interactive role in the intergenerational transmission of obesity pathway. DTI parameters, including MD, result from the modeling of diffusion processes that are non-specific to the cellular processes that underlie signal variation [48]. Thus, there are several biophysical explanations of variation in HTH MD including gliosis and/or reduced axonal outgrowth. Despite efforts to minimize the influence of partial volume effects (thresholded mask and median statistics), because of the relatively small size of the hypothalamus there is the inevitable possibility that extrahypothalamic structures may contaminate the ROI-based signal used here. Thus, future studies would benefit from increased spatial resolution [49] to further minimize partial voluming and potentially isolate effects to sub-nuclei within the hypothalamus and in proximity to the median eminence and the wall of the third ventricle. In addition, while several preclinical models have identified aberrant cellular processes within the developing hypothalamus that occur in offspring exposed to maternal overnutrition during pregnancy, to our knowledge, none of these models has attempted to synthesize these findings with the macroscopic approaches available for in vivo human observation (e.g., DTI). Thus, future translational efforts aimed at synthesizing detailed microscopy outcomes with in vivo methods that scale to humans would be of high scientific value. Finally, due to a lack of longitudinal data extending into early childhood across study populations at both sites, we were unable to characterize the prospective relevance of the newborn HTH MD for subsequent child overweight/obesity phenotypes. We note that we have previously reported an association between the HTH MD neurophenotype and early childhood body fat percentage in the study population at site 2 [21]. However, to validate the long-term functional relevance of variation in newborn human HTH MD, it is important that large scale longitudinal imaging efforts such as the HEALthy Brain and Child Development Study (HBCD) [50] are leveraged for this and similar purposes.

## Conclusions

In conclusion, the findings from the current study represent a significant conceptual advance to the literature by providing translational evidence of a robust association between maternal pre-pregnancy BMI and newborn offspring hypothalamic microstructure (consistent with preclinical animal findings). Importantly, because maternal BMI is potentially modifiable (at least in the short term) through diet, exercise [51], and/or a glucagon-like peptide-1 receptor agonist [52] (e.g., semaglutide), this work holds promise in contributing to an improved understanding of plausible intervention targets to prevent or attenuate the intergenerational transfer of obesity risk. Further, because such interventions may be limited in scope and effectiveness if initiated only after the occurrence of pregnancy [53], this work supports the emphasis on maternal pre-conceptional health in the context of offspring obesity risk.

## Supplementary Information

**Additional file 1: Table S1.** More Parsimonious Model Site Comparison: Maternal Pre-pregnancy BMI and Offspring Hypothalamic (HTH) Mean Diffusivity (MD). Maternal Pre-pregnancy BMI is associated with offspring HTH MD in a more parsimonious model excluding adjustment for white matter mean diffusivity. **Table S2.** Parsimonious Model With Sex Interaction Site Comparison: Maternal Pre-pregnancy BMI and Offspring Hypothalamic (HTH) Mean Diffusivity (MD). No evidence for a sex-specific association between maternal pre-pregnancy BMI and offspring infant HTH MD at either site (*p* > 0.1).

## Data Availability

Guidelines for strengthening the reporting of observational studies in epidemiology (STROBE, https://www.strobe-statement.org/index.php?id=available-checklists) were followed. Further, in accordance with the Material Design Analysis Reporting (MDAR) guidelines promoting access to underlying data and code, de-identified and unprocessed MRI data for site 2 used in the context of this study are publicly available through the National Institute of Mental Health Data Archive Collection #1890 (https://nda.nih.gov/edit_collection.html?id=1890). Unprocessed MRI data for site 1, de-identified pre-pregnancy BMI, and associated (e.g., covariates) measures can be made available upon request and by completing a Data Use Agreement. Derived measures and statistical models used here are made publicly available via a Github repository (https://github.com/jerodras/bmi-hypothalamus.git).

## Abbreviations

HTH: Hypothalamus
MD: Mean diffusivity
sFFA: Saturated free fatty acid
DTI: Diffusion tensor imaging
BMI: Body mass index
EPI: Echo planar imaging
TR: Repetition time
TE: Echo time
QC: Quality control
FA: Fractional anisotropy
WM: White matter
GA: Gestational age
FD: Framewise displacement
SA: Scan age
SD: Standard deviation
CNS: Central nervous system
OB: Obstetric
