# Supplementary material for: Maternal pre-pregnancy body mass index is associated with newborn offspring hypothalamic mean diffusivity: a prospective dual-cohort study
Source: BMC Med. 2023 Feb 14;21:57. doi: 10.1186/s12916-023-02743-8 (PMC9930241; doi:10.1186/s12916-023-02743-8)
Supplement: Supplementary file 1 — Additional file 1: Table S1. More Parsimonious Model Site Comparison: Maternal Pre-pregnancy BMI and Offspring Hypothalamic (HTH) Mean Diffusivity (MD). Maternal Pre-pregnancy BMI is associated with offspring HTH MD in a more parsimonious model excluding adjustment for white matter mean diffusivity. Table S2. Parsimonious Model With Sex Interaction Site Comparison: Maternal Pre-pregnancy BMI and Offspring Hypothalamic (HTH) Mean Diffusivity (MD). No evidence for a sex-specific association between maternal pre-pregnancy BMI and offspring infant HTH MD at either site (p > 0.1). [file 12916_2023_2743_MOESM1_ESM.docx]

**Additional File 1**

**Maternal Pre-pregnancy Body Mass Index is Associated with Newborn Offspring Hypothalamic Mean Diffusivity: A Prospective Dual-Cohort Study**

| **Maternal pBMI and Offspring Hypothalamic (HTH) Mean Diffusivity (MD): Parsimonious Model** | | | | | | | |  |
| --- | --- | --- | --- | --- | --- | --- | --- | --- |
|  |  | **Site 1 (N=152)** | | | | **Site 2 (N=79)** | |  |
| **Independent Variable** |  | $\hat{\boldsymbol{\beta}}$ | ***p*** |  | $\hat{\boldsymbol{\beta}}$ | | ***p*** | |
| Maternal pre-preg. BMI |  | 0.20 | *0.013* |  | 0.25 | | *0.029* | |
| Gestational Age at Birth |  | -0.16 | 0.100 |  | -0.20 | | 0.090 | |
| Postnatal Age at Scan |  | 0.11 | 0.250 |  | -0.17 | | 0.135 | |
| Infant Sex (Male) |  | -0.34 | *0.030* |  | 0.37 | | 0.155 | |

**Additional File 1: Table S1. More Parsimonious Model Site Comparison: Maternal Pre-pregnancy BMI and Offspring Hypothalamic (HTH) Mean Diffusivity (MD).** The more parsimonious model excludes adjustment for white matter mean diffusivity. na = not applicable, the data were denoised by removing images with motion, see methods section for details. Abbreviations used: BMI=Body mass index, MD=Mean diffusivity, QC=Quality control.

| **Maternal pBMI and Offspring Hypothalamic (HTH) Mean Diffusivity (MD): Parsimonious Model With Sex Interaction** | | | | | | | |  |
| --- | --- | --- | --- | --- | --- | --- | --- | --- |
|  |  | **Site 1 (N=152)** | | | | **Site 2 (N=79)** | |  |
| **Independent Variable** |  | $\hat{\boldsymbol{\beta}}$ | ***p*** |  | $\hat{\boldsymbol{\beta}}$ | | ***p*** | |
| Maternal pre-preg. BMI |  | 0.13 | *0.518* |  | 0.23 | | 0.133 | |
| Gestational Age at Birth |  | 0.19 | *0.042* |  | 0.19 | | 0.195 | |
| Postnatal Age at Scan |  | 0.45 | *<0.001* |  | 0.32 | | 0.065 | |
| Infant Sex (Male) |  | -0.22 | 0.801 |  | 0.37 | | 0.155 | |
| Sex x Maternal pre-preg. BMI |  | 0.00 | 0.999 |  | -0.01 | | 0.948 | |
| White Matter MD |  | 0.65 | *<0.001* |  | 0.69 | | *<0.001* | |
| QC (framewise displacement) |  | na | na |  | 0.20 | | 0.044 | |

**Additional File 1: Table S2. Parsimonious Model With Sex Interaction Site Comparison: Maternal Pre-pregnancy BMI and Offspring Hypothalamic (HTH) Mean Diffusivity (MD).** na = not applicable, the data were denoised by removing images with motion, see methods section for details. Abbreviations used: BMI=Body mass index, MD=Mean diffusivity, QC=Quality control.
